# Postoperative cognitive dysfunction—current research progress

**DOI:** 10.3389/fnbeh.2024.1328790

**Published:** 2024-01-30

**Authors:** Qi Zhao, Hui Wan, Hui Pan, Yiquan Xu

**Affiliations:** Department of Anesthesiology, Sichuan Cancer Hospital & Institute, Sichuan Cancer Center, Affiliated Cancer Hospital of University of Electronic Science and Technology of China, Chengdu, China

**Keywords:** POCD, review, risk factors, pathogenesis, prevention

## Abstract

Postoperative cognitive dysfunction (POCD) commonly occurs after surgery, particularly in elderly individuals. It is characterized by a notable decline in cognitive performance, encompassing memory, attention, coordination, orientation, verbal fluency, and executive function. This reduction in cognitive abilities contributes to extended hospital stays and heightened mortality. The prevalence of POCD can reach 40% within 1 week following cardiovascular surgery and remains as high as 17% 3 months post-surgery. Furthermore, POCD exacerbates the long-term risk of Alzheimer’s disease (AD). As a result, numerous studies have been conducted to investigate the molecular mechanisms underlying POCD and potential preventive strategies. This article provides a review of the research progress on POCD.

## 1 Introduction

Postoperative cognitive dysfunction (POCD) denotes a decline in neurocognitive function following anesthesia and surgical procedures, constituting a complication of the central nervous system (Rundshagen, 2014). Typically observed in individuals aged 65 and older (Evered and Silbert, 2018), POCD manifests primarily as reduced memory, attention, language fluency, orientation, and social skills after surgery (Needham et al., 2017). Since its inception (Rundshagen, 2014), substantial research has been conducted on POCD; however, a universally acknowledged specific pathogenesis remains elusive. Currently, it is widely accepted that POCD arises from the confluence of various factors, including patient age, surgical type, anesthesia modality, and pain intensity (Mason et al., 2010; Miller et al., 2018; Skvarc et al., 2018; Gu et al., 2019; Xiao et al., 2020). Moreover, studies indicate that POCD may persist for weeks to years, impacting patient recovery, prolonging hospitalization, and potentially leading to additional physical and mental ailments, heightened mortality, and significant burdens on patients and their families (Steinmetz et al., 2009). The ongoing trend of population aging, coupled with economic development, poses a challenge for anesthesia surgery, with an increasingly aged patient population. As patients undergoing surgery grow older, the likelihood of developing postoperative cognitive dysfunction rises. Consequently, addressing the identification and prevention of POCD has become a paramount concern. This article reviews POCD, encompassing epidemiology, risk factors, pathogenesis, and potential prevention strategies, while offering insights into future prospects for POCD prevention and treatment.

## 2 Definition and diagnostic criteria of POCD

Postoperative cognitive dysfunction (POCD), as per the cognitive impairment classification in the DSM-V, is characterized as a mild neurological disorder resulting from routine surgical procedures, excluding conditions like deafness, dementia, or amnesia (Edition, 2013). POCD entails a protracted cognitive decline lasting weeks, months, or even years and can be mistakenly conflated with postoperative delirium, presenting as an acute, fluctuating disturbance of consciousness typically within 1–3 days post-surgery (Steinmetz et al., 2009; Whitlock et al., 2011).

The diagnosis of POCD primarily relies on neurocognitive function scales, with widely used assessments including the Montreal Cognitive Assessment (MoCA), Wechsler Memory Scale (WMS), Wechsler Adult Intelligence Scale (WAIS), and Mini-Mental State Examination (MMSE) (Bouman et al., 2014; Chi et al., 2017; Pinto et al., 2019; Elkana et al., 2020). To mitigate biases stemming from external factors such as cultural backgrounds, education levels, and environmental or emotional states, multiple scales are often employed in combination when evaluating patients’ neurocognitive function (Ghoneim and Block, 2012; Needham et al., 2017). Following neurocognitive function evaluation, the diagnosis of POCD is commonly determined using methods like the SD method or Z-score method. The Z-score method involves selecting healthy elderly individuals for a baseline test, performing the test again after 1 week, and obtaining the mean ± standard deviation of the difference value between the two tests for healthy elderly individuals, defined as the “learning effect.” The patients’ preoperative scores are then subtracted from the postoperative scores, adjusting for the learning effect. The absolute value of the result is divided by the standard deviation of the healthy group to calculate the Z value ( $L=\frac{\sum \left(s2-s1\right)}{n}$ , L means the learning effect, s1 means the first test points in healthy people, s2 means the test points in healthy people after 1 week, *n* means the number of the including people; $SD=\sqrt{\frac{1}{n}\overset{n}{\underset{i=1}{\sum }}{\left(L_{i}-L\right)}^{2}}$ , $Z-\mathrm{value}=\frac{\left|S2-S1-L\right|}{SD}$ , S1 means the test scores in patients preoperative, S2 means the test scores in patients postoperative, SD means the standard deviation)When a patient has 2 or more Z-values greater than 1.96, the patient is considered to occur POCD (Moller et al., 1998). SD method: First, the preoperative scores of all patients included in the study are calculated to obtain the mean ± standard deviation, $(\bar{S}=\frac{\sum_{i=1}^{n}S_{i}}{n}$ , $SD=\sqrt{\frac{1}{n}\overset{n}{\underset{i=1}{\sum }}{\left(S_{i}-\bar{S}\right)}^{2}}$ , S means the test scores in patients preoperative, *n* means the number of the including patients). After subtracting the preoperative scores from the postoperative scores, the absolute value of the result is calculated. Postoperative deficits are identified as negative change values exceeding the standard deviation. In this context, a patient is deemed to have experienced POCD if they exhibit two or more neurocognitive test deficits (Saleh et al., 2015).

## 3 Epidemiology of POCD

POCD is prevalent among patients following cardiac or orthopedic surgery (Belrose and Noppens, 2019). Specifically, in individuals undergoing coronary artery bypass grafting and cardiopulmonary bypass, 50–70% develop POCD 1 week post-surgery (O'Gara et al., 2020). Furthermore, 10–30% experience long-term cognitive function effects 6 months after the procedure (Ghaffary et al., 2017). Among those undergoing hip arthroplasty, 20–50% encounter POCD within 1 week after surgery (Zhu et al., 2014; Li et al., 2019), with 10–14% still exhibiting POCD 3 months post-surgery (Krenk et al., 2014; Konishi et al., 2018).

Moreover, POCD is more prevalent in elderly patients, and its incidence increases with age (Moller et al., 1998). Studies indicate that, in non-cardiac surgery patients, those over 60 years old have a 1.5 times higher incidence of POCD compared to their younger counterparts. Additionally, the duration of POCD is longer in elderly patients than in younger ones (Monk et al., 2008).

## 4 Risk factors of POCD

### 4.1 Age

In clinical settings, the aging trend of surgical patients is evident, resulting in a relatively high proportion of elderly patients experiencing POCD post-surgery (Moller et al., 1998). Research indicates that around 30% of younger patients and approximately 40% of older patients develop POCD upon hospital discharge. Notably, 12.7% of elderly patients remain in POCD 3 months after surgery, while only 5% of their younger counterparts persist in POCD (Monk et al., 2008). The physiological decline in elderly patients, coupled with potential respiratory, cardiovascular, and cerebrovascular diseases, significantly diminishes their tolerance to anesthesia (Dodds and Allison, 1998; Ancelin et al., 2010). Consequently, the incidence of POCD is markedly higher in elderly patients.

### 4.2 Types of surgery

Osteoarthritis is a prevalent joint ailment in elderly patients, often addressed through hip and knee arthroplasty procedures (Abramoff and Caldera, 2020). Despite the maturity of these surgeries in enhancing joint function and alleviating pain (Canovas and Dagneaux, 2018; Lu and Phillips, 2019), the risk of POCD remains elevated compared to general surgery. This heightened risk is attributed to prolonged surgery durations, increased surgical trauma, and the potential occurrence of fat embolism (Krenk et al., 2014).

Coronary artery disease is a highly lethal condition worldwide, and its incidence and fatality rates have risen recently. Coronary artery bypass grafting is widely acknowledged as the most efficient treatment for coronary artery disease; however, it is associated with an elevated risk of POCD, particularly following coronary artery bypass grafting and cardiopulmonary bypass (CPB) (Wan et al., 2020). POCD primarily stems from inadequate perfusion during cardiac surgery, cerebral microembolism, and inflammation (Pappa et al., 2017). Within the CPB process, the cardiopulmonary bypass machine circulates microemboli like air bubbles, fat, and blood clots into the brain. Additionally, clamping the aorta during the procedure may dislodge atherosclerotic plaques, leading to embolism. A study observed minute emboli in the brains of patients who succumbed after undergoing coronary artery bypass grafting with cardiopulmonary bypass (CABG-CPB) (Moody et al., 1995). Head MRI studies have verified that around 50% of CABG recipients undergo brain microembolic infarction, resulting in mild neurological impairment (Knipp et al., 2008). It is confirmed that the immune system is activated during CPB when blood components interact with artificial materials. This activation triggers widespread systemic inflammatory responses, causing reperfusion injury to organs. Furthermore, peripheral inflammatory factors breach the blood–brain barrier, inducing central nervous system inflammation and POCD (Day and Taylor, 2005).

Table 1 compare the incidence of POCD in various age groups following cardiac and non-cardiac surgeries. Due to a lower frequency of cardiac surgeries in younger individuals, Table 2 exclusively presents the morbidity of POCD in elderly patients.

**Table 1:** Morbidity in age-matched patients (non-cardiac surgery) (Monk et al., 2008).

Time point	Morbidity in young patients	Morbidity in middle-age patients	Morbidity in elderly patients
At hospital discharge	36.6%	30.4%	41.4%
At 3 months after surgery	5.7%	5.6%	12.7%

**Table 2:** Morbidity in elderly patients (cardiac surgery) (Ghaffary et al., 2017).

Time point	Morbidity in young patients	Morbidity in middle-age patients	Morbidity in elderly patients
At hospital discharge	–	–	50–80%
At 6 months after surgery	–	–	10–30%

### 4.3 Types of anesthesia

According to the surgical requirements, the anesthesiologist will opt for either intravertebral anesthesia or general anesthesia. Previous research indicates that there is no discernible difference in the occurrence of POCD between knee arthroplasty patients undergoing either general or intravertebral anesthesia 1 week and 6 months post-surgery (Williams-Russo et al., 1995). In a study, patients undergoing hip arthroplasty were randomly assigned to three groups receiving sedation with midazolam, dexmedetomidine, or propofol under spinal-epidural block. The incidence of POCD in the propofol group 1 week after surgery was notably lower than in the midazolam and dexmedetomidine groups (Li et al., 2019). The combination of midazolam and isoflurane is a widely employed anesthesia approach. An investigation utilizing this combination for rat anesthesia revealed widespread apoptosis of hippocampal neurons and dysfunction in hippocampal synapses, potentially contributing to cognitive function decline (Jevtovic-Todorovic et al., 2003). A meta-analysis of 28 Randomized Controlled Trials (RCTs) concluded that the incidence of POCD is lower in surgeries maintaining intravenous anesthesia with propofol compared to those using inhalation anesthesia with isoflurane or sevoflurane (Miller et al., 2018).

### 4.4 Pain management

After surgery, postoperative pain is a common issue, mainly stemming from substantial surgical trauma or the potential for wound infection. Consequently, prophylactic antibiotics are administered following certain operations, and patients are equipped with analgesia pumps to alleviate pain. A study revealed that utilizing a postoperative patient-controlled analgesia pump independently increased the risk of POCD. In comparison to those who received intravenous opioids through a patient-controlled analgesia device, individuals who orally received postoperative analgesia faced a significantly lower risk of developing POCD (Wang et al., 2007). Su et al. demonstrated that incorporating dexmedetomidine as an adjunct, as opposed to routine postoperative analgesia, could reduce pain and enhance postoperative cognitive function (Su et al., 2022). Additionally, meta-analyses on pain indicate that persistent pain may lead to a decline in patients’ cognitive abilities, attention, memory, and information processing (Berryman et al., 2013; Mazza et al., 2018).

### 4.5 Education level

Individuals with higher education levels may experience a lower occurrence of POCD. In this educated demographic, the brain consistently engages in challenging intellectual tasks, bolstering neuronal reserve and synaptic function. In instances of surgical trauma, a robust brain reserve could mitigate hippocampal damage, consequently diminishing cognitive decline post-surgery (Kotekar et al., 2018). Higher-educated patients exhibit enhanced tolerance to pathological changes induced by neurodegenerative diseases. Additionally, those with greater cultural reserve demonstrate a deceleration in the aging process and cognitive function decline (Stern, 2012).

## 5 Pathogenesis of POCD

### 5.1 Central inflammation

The emergence of POCD is now associated with the inflammatory response within the central nervous system. Studies affirm that, following surgical trauma, peripheral inflammatory factors breach the blood–brain barrier (BBB), causing central nervous system inflammation (Alam et al., 2018; Zhu et al., 2018). Cibelli et al. observed that IL-1 receptor knockout mice, subjected to tibial fracture, maintained unimpaired cognitive function with no increase in IL-1β levels. They proposed that surgery-induced innate immune responses activate IL-1β-mediated inflammatory reactions in the hippocampus, resulting in cognitive impairment (Cibelli et al., 2010). Feng et al. demonstrated that mice with depleted microglia did not develop POCD after tibial fracture, and hippocampal inflammation remained unaltered. This suggests that surgery-induced microglia activation contributes to hippocampal inflammation, with activated microglia promoting the entry of peripheral inflammatory factors into the nervous system and exacerbating inflammation levels (Feng et al., 2017). Bowman et al. hypothesized that BBB injury induces POCD in patients. Those with BBB injury exhibited a generally increased concentration of inflammatory factors in cerebrospinal fluid, strongly implicating nervous system inflammation in the genesis of POCD (Bowman et al., 2018).

### 5.2 Neuronal apoptosis

Neurons form the foundation of cognitive function. Cognitive decline, memory loss, diminished learning abilities, and emotional changes in patients can result from brain damage caused by neuronal apoptosis. While neurons in the adult brain possess a self-healing capacity through regeneration, aging diminishes this ability (Vasic et al., 2019). Sun et al. observed a significantly higher apoptosis index of hippocampal neurons in AD mice compared to the wild-type control group, as indicated by TUNEL staining. Treatment of AD mice with dexmedetomidine led to a reduction in hippocampal neuronal apoptosis and an improvement in cognitive function (Sun et al., 2020). Yuan et al., in their POCD model using aged mice with the left extrahepatic lobe resected, proposed that the activation of the NF-κB pathway in neurons triggers neuronal apoptosis and autophagy, resulting in cognitive impairment (Yuan et al., 2022). Shao et al. induced the POCD model through sevoflurane and discovered that Chikusetsu saponin IVa (ChIV) exerts a neuroprotective effect against sevoflurane-induced neuroinflammation and cognitive impairment by inhibiting the NLRP3/caspase-1 pathway. This suggests that ChIV could be a promising clinical strategy for treating anesthesia-induced POCD in elderly patients (Shao et al., 2020).

### 5.3 Synaptic plasticity impairment

Synaptic plasticity denotes the autonomous adjustment of connection strength between nerve cell synapses, leading to short-term (inhibition, facilitation, enhancement) and long-term (long-term potentiation, long-term inhibition) changes in synaptic morphology and function (Magee and Grienberger, 2020). This phenomenon forms the neural basis for memory and learning mechanisms (Martin et al., 2000; Magee and Grienberger, 2020). Post-synaptic density protein 95 (PSD95) plays a crucial role in synaptic plasticity. Gao et al. observed that surgical and anesthesia stimulation led to a reduction in synaptophysin and PSD95 in the mouse hippocampus, impairing postoperative cognitive function (Gao et al., 2021). In a POCD model induced by exploratory laparotomy under anesthesia in aged mice, Li et al. identified increased expression of histone deacetylase 3 in the dorsal hippocampus, accompanied by significantly decreased levels of dendritic spine density and synaptic plasticity-related proteins. Selective blockade of histone deacetylase 3 in the dorsal hippocampus restored dendritic spine density and synaptic plasticity-related proteins, resulting in the reversal of postoperative cognitive decline (Yang et al., 2022). Similarly, Luo et al. discovered that surgery elevated the activity of histone deacetylase 2 and decreased dendritic arborization and spine density in the hippocampus of POCD mice. Intraperitoneal injection of suberanilohydroxamic acid, a relatively specific inhibitor of histone deacetylase 2, reversed these surgery-induced effects (Luo et al., 2020). Xiao et al. reported that inhibiting prostaglandin E2-receptor3 through dorsal hippocampus stereotaxic virus injection rescued the expression of plasticity-related proteins, including cAMP response element-binding protein and activity-regulated cytoskeletal-associated protein, alleviating surgery-induced cognitive decline (Xiao et al., 2018).

### 5.4 Abnormal modification of tau protein

Tau protein, a key member of the tubulin family, plays a crucial role in microtubule assembly and forms the structural foundation of the neural cytoskeleton (Ukmar-Godec et al., 2020). In physiological conditions, Tau protein undergoes a dynamic balance of phosphorylation and dephosphorylation, maintaining the stability of the neural cytoskeleton structure. Hyperphosphorylation of Tau protein results in the formation of neurofibrillary tangles, triggering neuronal apoptosis and contributing to neurodegenerative diseases (Avila et al., 2004; Pîrşcoveanu et al., 2017). Yan et al. observed a significant increase in phosphorylated Tau protein levels in the hippocampal tissue and cultured neurons of an aged POCD mouse model (Yan et al., 2020). Mattsson et al. established a correlation between the levels of total tau protein and phosphorylated tau protein in cerebrospinal fluid and the disease state of AD. These proteins can serve as biomarkers predicting the onset of AD (Mattsson et al., 2017). Dai et al. isolated phosphorylated Tau protein from the brains of deceased AD patients and injected it into the hippocampus of AD mice. In half of the mice, monoclonal antibody 43D was administered intravenously to eliminate pathological tau protein. Treatment with the 43D monoclonal antibody effectively hindered the abnormal modification and aggregation of Tau protein, blocking its spread to the contralateral hippocampus. This finding suggests that phosphorylated Tau protein may play a crucial role in the progression of AD (Dai et al., 2018).

### 5.5 Pain

Pain, a multifaceted physiological and psychological experience, is a prevalent clinical symptom with potential links to the onset of POCD. Berryman et al. discovered that individuals experiencing chronic pain exhibited poorer performance on working memory tests compared to healthy controls (Berryman et al., 2013). Simultaneously, executive abilities were also observed to be diminished in patients with chronic pain compared to their healthy counterparts (Berryman et al., 2014). Liu et al. identified severe memory impairment in patients with chronic pain, suggesting that pain might induce distraction and reduce memory capacity (Liu et al., 2014, 2021). Veldhuijzen et al. noted impaired problem-solving skills and decreased speed in individuals with fibromyalgia (Veldhuijzen et al., 2012). Establishing a rat model of sciatic nerve chronic constriction injury (CCI), Zhang et al. observed the development of POCD persisting for at least 21 days post-surgery in the CCI group (Zhang et al., 2021). Ding et al. reported compromised learning and memory abilities in a mouse model of sciatic nerve CCI, suggesting that CCI-induced chronic pain might exacerbate POCD through alterations in neurotransmitter levels and inflammatory factors (Ding et al., 2021). Pain significantly influences various neurodegenerative diseases, including AD, often underestimated due to communication challenges faced by AD patients (Cravello et al., 2019). Notably, pain levels in AD patients positively correlate with disease severity (Scherder et al., 2008; Whitlock et al., 2017). Shared pathological mechanisms, such as abnormal noradrenergic system function, microglial activation in the frontal cortex, and heightened inflammation, underlie both AD and pain (Salter and Stevens, 2017; Hayashida and Obata, 2019). Chronic pain and cognitive decline share intertwined pathological pathways, with the progression of pain intricately linked to the development of cognitive impairment.

### 5.6 Mitochondrial metabolic disorder

Aging, a natural biological process, is marked by the decline of bioenergy (López-Otín et al., 2013). Mitochondrial metabolism, a central process in bodily capacitation, is closely linked to diseases associated with aging (Natarajan et al., 2020). Yang et al. observed increased mitochondrial fragmentation in the hippocampus of POCD mice, linking neuroinflammation to mitochondrial dysfunction in primary cells. Enhancing mitochondrial function effectively improved postoperative cognitive function in mice (Yang et al., 2021). In a rat model of POCD, Netto et al. identified neuroinflammation and oxidative stress as sources of free radicals that activate the central nervous immune system, impair mitochondrial function, influence the production of brain-derived neurotrophic factor (BDNF), and contribute to the occurrence of POCD (Netto et al., 2018). Han et al. demonstrated that high expression of PGC-1α in the hippocampus improves cognitive function in mice. This increase in expression enhances mitochondrial antioxidant activity, inhibits neuroinflammation and reactive oxygen species production, boosts neuronal metabolic activity, and reduces mitochondrial damage, presenting a potential target for POCD treatment (Han et al., 2020).

### 5.7 Neurotrophic factor

Brain-derived neurotrophic factor (BDNF) exerts neurotrophic effects, with widespread expression of BDNF and its receptors in the central nervous system. Their primary role involves enhancing synaptic plasticity and promoting neurogenesis in the hippocampus, crucial for brain learning and memory function (Lu et al., 2013). In patients with vascular cognitive impairment, Wang et al. identified reduced BDNF expression in the serum. Cell experiments revealed that negative regulation of BDNF expression triggered nerve cell apoptosis, leading to cognitive impairment (Wang et al., 2020). Qiu et al. observed that anesthesia-induced neuroinflammation resulted in dysregulation of the BDNF/TrkB signaling pathway, causing dendritic spine loss and apoptosis, leading to POCD in mice (Qiu et al., 2020). Amidfar et al. found that beta-amyloid inhibits BDNF expression, contributing to cognitive decline (Amidfar et al., 2020). Successfully constructing a dementia model through entorhinal cortex synapse damage, Ando et al. overexpressed the BDNF gene in the entorhinal cortex, demonstrating that high BDNF expression could reverse cognitive decline (Ando et al., 2002).

## 6 Prevention of POCD

Current research indicates that POCD results from various factors, with hospitals generally lacking awareness of early prevention strategies for the condition. Therefore, early prevention emerges as a crucial strategy to reduce the incidence of POCD.

### 6.1 Identify biomarkers

Early prediction of POCD is pivotal for proactively addressing potential issues and formulating preventive plans. Duan et al. discovered that serum glial cell line-derived neurotrophic factor (GDNF) serves as a predictive biomarker for POCD diagnosis. ΔGDNF on the second day postoperatively demonstrated early predictive efficacy for POCD, with a threshold set at 49.1 (Duan et al., 2018). Liu et al. established a link between the occurrence of POCD and the concentration of peripheral inflammatory factors, identifying C-reactive protein and IL-6 as the most significant markers in this context (Liu et al., 2018). Given the variety of studies on POCD-related biomarkers, selecting appropriate biomarkers can enhance predictive accuracy for the onset of POCD.

### 6.2 Cognitive function training

Saleh et al. demonstrated that preoperative cognitive function training, such as the Method of Loci (MoL), could effectively prevent the occurrence of POCD (Saleh et al., 2015). O’Gara et al. employed a mobile application to train patients in memory, attention, problem-solving, flexibility, and processing speed until 1 month post-surgery. Although there was no significant difference in the incidence of POCD between the cognitive training group and the routine nursing group, it was noted that the complexity of the training might have reduced patient compliance. However, the cognitive training group exhibited significantly better memory and cognitive abilities than the routine nursing group. Hence, there is a need for further development and design of appropriate cognitive training programs to maximize benefits (O'Gara et al., 2020). Humeidan et al. utilized electronic tablets for preoperative cognitive exercises targeting memory, speed, attention, flexibility, and problem-solving functions. The intervention was found to reduce the risk of delirium in patients who were minimally compliant at the very least (Humeidan et al., 2021).

### 6.3 Rational use of drug in the perioperative period

Liang et al. demonstrated that perioperative administration of ulinastatin could reduce postoperative inflammation levels and lower the incidence of POCD (Lv et al., 2016; Liang et al., 2021). Wang et al. found that patients receiving lidocaine exhibited a reduced occurrence of POCD, potentially attributable to lidocaine’s neuroprotective effects (Wang et al., 2002). Similarly, Chen et al. reported the effectiveness of continuous lidocaine infusion during surgery in reducing POCD incidence among elderly patients undergoing spinal surgery (Chen et al., 2015). However, Mathew et al. found no significant difference in POCD incidence between the lidocaine treatment group and the placebo treatment group (Mathew et al., 2009). Su et al. observed that prophylactic low-dose dexmedetomidine during the perioperative period resulted in a lower incidence of postoperative delirium and fewer adverse cardiovascular events, affirming the safety and efficacy of dexmedetomidine in preventing cognitive decline (Su et al., 2016). Conversely, Deiner et al. reported that intraoperative dexmedetomidine did not reduce the incidence of POCD (Deiner et al., 2017). Valentin et al. found that intravenous dexamethasone administered before general anesthesia effectively reduced the incidence of POCD, particularly in the memory and executive cognitive domains (Valentin et al., 2016). Papadopoulos et al. discovered that postoperative administration of anstenchon could reduce the incidence of POCD after hip arthroplasty, providing relief from patient pain (Papadopoulos et al., 2014). It is evident that current research on perioperative medication to reduce the incidence of POCD remains controversial, as the drug concentration, timing of use, and duration of administration all play crucial roles.

### 6.4 Multidisciplinary collaboration

Multidisciplinary collaboration holds significant value in preventing POCD. The synergy between anesthesiologists and surgeons fosters effective communication and cooperation, aiding in the selection of optimal anesthesia and surgical plans. This collaborative effort enables cautious use of anesthesia drugs, optimization of patient admission and operation times, and reduction of postoperative complications. Timely communication between anesthesiologists and surgical departments enhances postoperative pain management, nutrition management, and rehabilitation, contributing to improved patient outcomes. Interaction between anesthesiologists and patients, along with their families, encourages swift reintegration into society. Family support helps alleviate post-surgery anxiety in patients, reducing the likelihood of POCD occurrence (Egerod et al., 2010; Laalou et al., 2011).

## 7 Discussion

POCD is a multifactorial neurodegenerative disease influenced by various factors, including patient age, surgery type, anesthesia plan, postoperative pain, and educational level, all of which contribute to the risk of POCD. This condition commonly manifests after cardiac and orthopedic surgeries, often persisting for weeks to months, with some cases enduring for several years, impacting the recovery process and increasing mortality rates.

While neurocognitive evaluation remains the optimal method for identifying POCD, patient compliance may sometimes be challenging. Consequently, the search for a highly specific and sensitive biomarker as a predictor of POCD becomes imperative. Identification of high-risk patients enables surgeons and anesthesiologists to exercise greater caution during surgery, minimizing the likelihood of POCD occurrence. Current research on POCD has delved into its pathogenesis, implicating factors such as central nervous system inflammation, neuronal apoptosis, synaptic plasticity damage, abnormal modification of Tau protein, chronic pain, and mitochondrial metabolic disorders. Several studies have confirmed the efficacy of certain drugs and cognitive training in preventing POCD. Appropriate and timely application of these interventions proves effective in reducing the incidence of POCD.

In contemporary research, the construction of the POCD model has reached a mature stage, employing various methods such as unilateral nephrectomy under anesthesia in aged mice (Liu et al., 2023), exploratory laparotomy under anesthesia in aged mice (Qiu et al., 2020), resection of the left extrahepatic lobe in aged mice (Yuan et al., 2022), and inducing a tibia fracture via orthopedic surgery in middle-aged rats (Garrone et al., 2021). Cognitive function assessment involves tests such as the fear conditioning test, open field test, Morris Water Maze test, Y-Maze test, among others. Through extensive animal experiments, scientists have identified various signal pathways associated with POCD, including the NF-κB pathway in central inflammation, the caspase protein family in neuronal apoptosis, and TLRs in inflammation and pain (Chen et al., 2019).

As of now, substantial progress has been made in understanding POCD, and effective methods for its prevention and treatment have been identified. The anticipation is that future research will delve further into POCD treatment. Drawing from the current knowledge of pathogenic pathways, targeted therapy drugs may be developed to address POCD. Potential targets include Tau protein, NF-κB pathway, caspase protein family, IL-1β, and Aβ. Such targeted therapies hold promise in safeguarding the well-being of patients affected by POCD.

## Author contributions

QZ: Funding acquisition, Writing – original draft, Writing – review & editing. HW: Software, Writing – original draft, Writing – review & editing. HP: Software, Writing – review & editing. YX: Funding acquisition, Writing – review & editing.
